# Infantile hypothyroidism and its relationship with delayed tooth eruption: A case report

**DOI:** 10.4317/jced.59587

**Published:** 2022-12-01

**Authors:** Isabella-Fernandes Carvalho, Arine-Alcoforado Amorim, Paulo-Tárcio-Aded da Silva, Maria-Denise-Fernandes-Carvalho de Andrade, Ellaine-Doris-Fernandes Carvalho, Rheryda-de Sousa-Rocha-Pereira Freitas, José-Higino-da Silva Neto, José-Luciano-Pimenta Couto

**Affiliations:** 1 Post-graduate program in Dental Sciences, Unichristus, Fortaleza, Brazil; 2 Graduate program in Medicine, Unichristus, Fortaleza, Brazil; 3 Graduate Dentistry, Unichristus, Fortaleza, Brazil; 4 Post-graduate program in Dental Sciences, Federal University of Sobral, Brazil

## Abstract

Hypothyroidism is characterized as a systemic endocrine disorder that is caused by a dysfunction of the thyroid gland. This produces the thyroid hormones T3 and T4 that are responsible for carrying out the normal functions of the physical body, that is, changes in the secretion of these hormones may be related to some maladjustments in the stomatognathic system. The most common manifestations of congenital hypothyroidism, also known as cretinism, are thick lips, macroglossia, malocclusion and delayed eruption of both dentitions. This study aims to report a case of a child with hypothyroidism and a delay in the chronology of tooth eruption. Patient, female, 9 years and 8 months old, whose main complaint was a delay in the chronology of tooth eruption. On clinical examination, a marked delay in the chronology of tooth eruption was observed. Therefore, it was necessary to refer the patient to a geneticist, who ruled out any syndromic alteration. Then, the patient was referred to the endocrinologist, whose opinion was hypothyroidism.

** Key words:**Hypothyroidism, tooth eruption, endocrine disorder, child health.

## Introduction

The endocrine system is responsible for the release of various hormones and is strongly interconnected with the central nervous system. In addition, it controls the physiological functioning of the body and is responsible for maintaining homeostasis. In recent years, the early diagnosis of systemic diseases such as metabolic disorders, autoimmune or hormonal diseases has had a higher prevalence due to an improvement in the population’s life expectancy (1).

The thyroid hormones T3 (triiodothyronia) and T4 (thyroxine) are produced by the thyroid gland and correspond to the main regulators of somatic metabolism. Such hormones act from the maintenance of body temperature, metabolism of proteins, lipids and vitamins, regulation of the local metabolic activity of the alveolar bone, to the potentiation of the action of other hormones. Thus, to maintain the normal activity of target tissues, adequate serum levels of these hormones must be guaranteed, which depends not only on thyroid activity, but also on the integrity of the hypothalamic-pituitary-thyroid axis (1-8).

Hypothyroidism is an endocrine disorder, of a systemic nature, characterized by dysfunction of the thyroid gland, which produces thyroid hormones below adequate serum levels. The frequency of hypothyroidism is higher among women and its incidence increases with advancing age. Its etiology may be related to different factors, among which iodine deficiency, tissue dysgenesis, absence of essential enzymes for hormone synthesis or autoimmune processes such as Hashimoto’s Thyroiditis stand out (9).

The most common manifestations of congenital hypothyroidism, also known as cretinism, are thick lips, macroglossia, malocclusion and delayed eruption of both dentitions. Lip thickening and tongue enlargement (macroglossia) is due to increased accumulation of smooth subcutaneous mucopolysaccharides, which are obtained as a result of the breakdown of glycosaminoglycans (10).

Few studies were found in the literature on patients with thyroid disorders, so the objective of this study is to report the clinical case of a child with hypothyroidism and delay in the chronology of tooth eruption, seen at the Dentistry service of Centro Universitário Christus.

Case Report

Patient, R.S.L, female, 9 years and 8 months old, arrived at the children’s clinic of Centro Universitário Christus accompanied by her mother, with the complaint of having a “delay in changing teeth and the presence of some cavities”. During the anamnesis, no relevant systemic impairment was reported.

**Figure 1:**
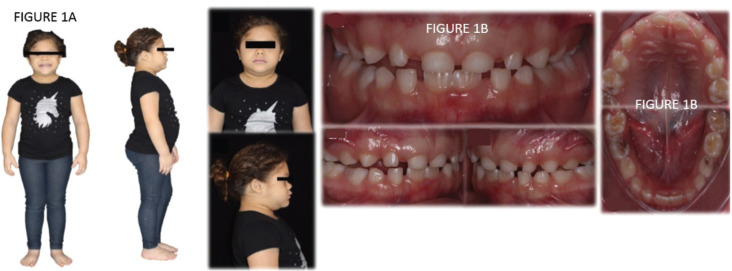
A) Photos of the patient’s body highlighting the short stature and the shortening of the lower and upper limbs. Frontal photo of the patient. Profile picture of the patient. Close-up extraoral photos of the patient. Front view of the patient. Profile view of the patient highlighting the fat protuberance of the submentum. B) Intraoral photos of the patient. Photo of the predominantly deciduous dentition, except for the lower central incisors. Photo in right lateral occlusion. Photo in left lateral occlusion. Occlusal photos of the patient. Superior Occlusal View. Inferior Occlusal View.

In the physical examination, a short stature of the patient was observed, with emphasis on the shortening of the upper and lower limbs and linked to this, an overweight (Fig. 1A).

In the intra-oral examination, there was a significant delay in the chronology of tooth eruption, with all deciduous teeth still present, except for the lower central incisors, which had already erupted the permanent successors, as well as the presence of the first permanent molars. Carious lesions were diagnosed on teeth 54, 64, 74 and 85 (Fig. 1B).

**Figure 2:**
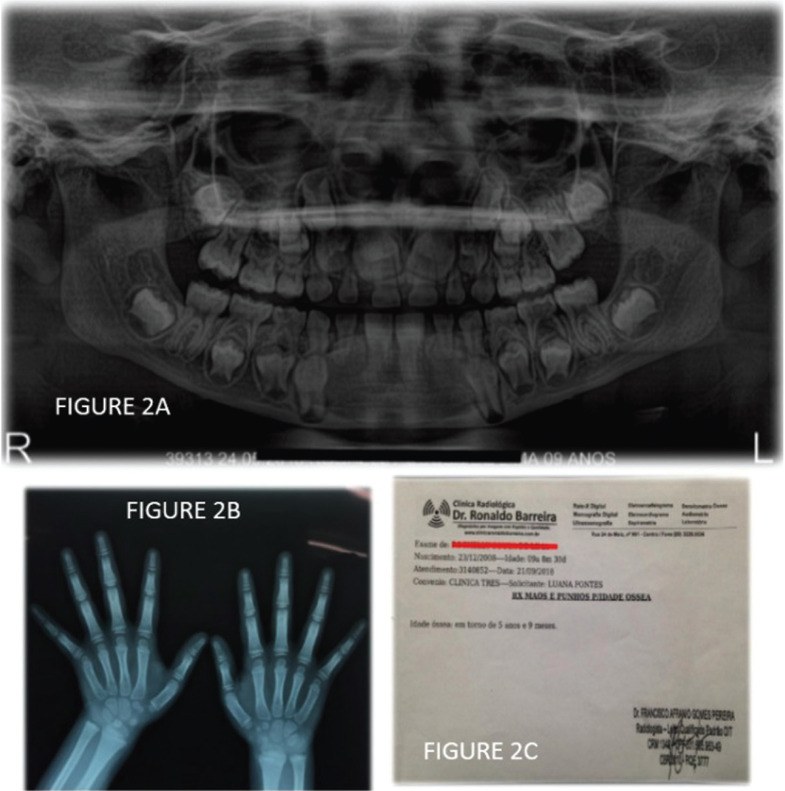
A) Panoramic radiograph of the patient. B) Radiograph of the patient’s hand and wrists. C) Hand and wrist radiography report showing that the bone age corresponds to 5 years and nine months.

The panoramic radiograph showed that there were no changes in the number of teeth (Fig. 2A). In addition, the roots of permanent teeth that had not yet erupted were in the process of formation. On the other hand, the roots of deciduous teeth showed only discrete traces of resorption, revealing a delay in the process of rooting. Hand and wrist radiography revealed the patient’s bone age equivalent to 5 years and 9 months, with a chronological age of 9 years and 8 months (Fig. 2B,C).

**Figure 3:**
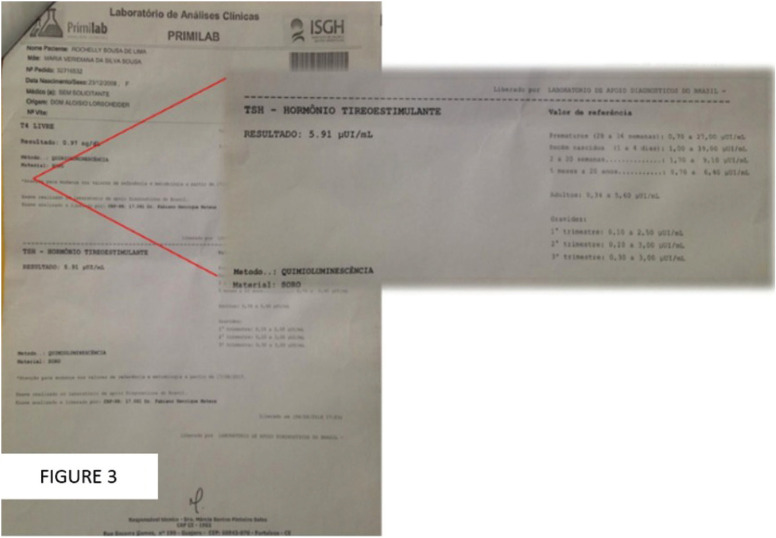
Patient’s report of serum TSH levels.

Given the scenario presented by the patient about her physical and oral characteristics, she was referred to a geneticist, who, through more specific tests, such as karyotype, ruled out any syndromic alteration. However, the geneticist then referred her to an endocrinologist, who, through the hormone dosage exam, diagnosed the patient with hypothyroidism (Fig. 3).

The proposed treatment was multidisciplinary, as initially there was a hormone replacement with levothyroxine sodium, in order to supply the deficit of T3 and T4 thyroid hormones.

**Figure 4:**
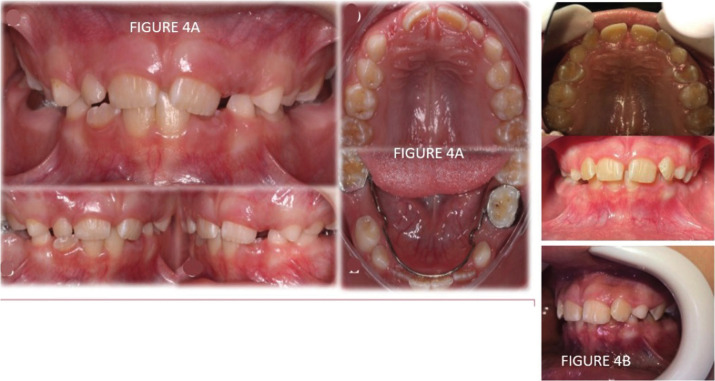
A) Intraoral photos of the patient after 6 months of treatment. Image in occlusion, birth of maxillary and mandibular central incisors. Right lateral photo. Left lateral photo . Superior occlusal view, upper lateral exfoliation. Inferior occlusal view, with space maintainer installed. B) left side view, superior occlusal view and front view.

The patient signed an informed consent form accepting the proposed dental treatment, which included: restorations in decayed teeth (54 and 64), extraction of the dental elements (85 and 75), in view of a great pulp involvement, and due to bilateral extractions. , we chose to install the Nance lingual arch space maintainer. The restorations in question were performed with resin-modified glass ionomer cement, due to its fluoride release and adequate esthetics (Fig. 4).

One year after the first visit, the patient returned and it was possible to verify the eruption of the teeth corresponding to the chronology of age (Fig. 4A,B).

## Discussion

Thyroid hormones are necessary to supply the body’s normal organic functions. Thus, patients in a state of hypothyroidism usually present as characteristic signs and symptoms weight gain, hypotension, cold, thick and rough skin, , muscle weakness with slow reflexes, lethargy, slow heart rate, swelling of the face and eyelids, non-dispersible swelling of the limbs (myxedema), mental retardation and swallowing problems (11,12).

Delay in skeletal maturation, confirmed by delayed appearance and growth of epiphyseal ossification centers, may also characterize these patients. In children, the absence of the distal femoral and proximal tibial epiphysis is an important radiographic finding. In young people, the abnormal maturation of the epiphysis leads to distinct radiographic findings, with irregular fragmented contours called epiphyseal dysgenesis (13).

Prolonged retention of deciduous teeth may occur in the absence of permanent successors (permanent canines and premolars); in the presence of excess permanent teeth (supernumerary), which prevent the formation of an eruption corridor for the successor; in ankylosis of deciduous teeth; in some hormonal changes, as in hypothyroidism; in severe forms of hypopituitarism; and in some syndromes, such as trisomy , cleidocranial dysplasia and Hurler Syndrome (9).

The process of tooth eruption is not fully understood, but it can be said that four mechanisms compose it in the permanent tooth: the formation of the tooth root, during which the growing root is accommodated through the intraosseous movement of the tooth crown and the root resorption of the deciduous predecessor; the hydrostatic pressure on the periapical tissues, pushing the tooth in the occlusal direction; bone remodeling and movement of the tooth in an occlusal direction by the cells and fibers of the periodontal ligament (14).

The patient in the present study was diagnosed with hypothyroidism associated with oral manifestations of prolonged retention of primary teeth and delay in the formation of roots of permanent teeth. The hand and wrist radiography report showed that her bone age corresponded to 5 years and nine months. Medical treatment consisted of hormone replacement with levothyroxine sodium, 25mcg, box of 30 Tablets, the ideal being 3 mcg/kg/day.

Levothyroxine is the mainstay of treatment for hypothyroidism, and is one of the World Health Organization’s essential medicines needed for basic health care. This has been the “gold standard” for thyroid hormone replacement therapy, in the exogenous form of T4, for over sixty years. The first report occurred in the 1890s when a thyroid gland was injected into a patient with myxoedema (15-17).

Levothyroxine is available in Tablets and softgels, intravenously and, more recently, in liquid formulations. Liquid formulations demonstrate improved absorption when taken with food (18).

The starting dose of levothyroxine depends on the patient’s age, presence of coexisting heart disease, etiology and severity of the patient’s biochemical hypothyroidism. The levothyroxine dose is titrated until TSH levels normalize between 0.4 and 4.0 mIU/L (19,20).

Thus, it was possible to observe that a good dental evaluation, concomitant with medical treatment, after diagnosis and immediate initiation of the patient’s hormonal treatment, led to an improved quality of life.
